# Telehealth-Based vs In-Person Aerobic Exercise in Individuals With Schizophrenia: Comparative Analysis of Feasibility, Safety, and Efficacy

**DOI:** 10.2196/68251

**Published:** 2025-02-14

**Authors:** David Kimhy, Luz H Ospina, Melanie Wall, Daniel M Alschuler, Lars F Jarskog, Jacob S Ballon, Joseph McEvoy, Matthew N Bartels, Richard Buchsbaum, Marianne Goodman, Sloane A Miller, T Scott Stroup

**Affiliations:** 1 Department of Psychiatry, Icahn School of Medicine at Mount Sinai, 1 Gustave L. Levy Place, Box 1230, New York, NY, 10029, United States, 1 212-585-4656; 2 New York Mental Illness Research Education and Clinical Center (NY MIRECC), James J. Peters VA Medical Center, New York, NY, United States; 3 Department of Psychiatry, Columbia University, New York, NY, United States; 4 Department of Psychiatry, University of North Carolina, Chapel Hill, NC, United States; 5 Department of Psychiatry and Behavioral Sciences, Stanford University, Stanford, CA, United States; 6 Department of Psychiatry and Health Behavior, Georgia Regents Medical Center, Augusta, GA, United States; 7 Department of Rehabilitation Medicine, Albert Einstein College of Medicine, Bronx, NY, United States

**Keywords:** schizophrenia, psychosis, exercise, aerobic fitness, VO_2_max, telehealth, telemedicine, COVID-19, clinical trial, safety, maximum oxygen consumption

## Abstract

**Background:**

Aerobic exercise (AE) training has been shown to enhance aerobic fitness in people with schizophrenia. Traditionally, such training has been administered in person at gyms or other communal exercise spaces. However, following the advent of the COVID-19 pandemic, many clinics transitioned their services to telehealth-based delivery. Yet, at present, there is scarce information about the feasibility, safety, and efficacy of telehealth-based AE in this population.

**Objective:**

To examine the feasibility, safety, and efficacy of trainer-led, at-home, telehealth-based AE in individuals with schizophrenia.

**Methods:**

We analyzed data from the AE arm (n=37) of a single-blind, randomized clinical trial examining the impact of a 12-week AE intervention in people with schizophrenia. Following the onset of the COVID-19 pandemic, the AE trial intervention transitioned from in-person to at-home, telehealth-based delivery of AE, with the training frequency and duration remaining identical. We compared the feasibility, safety, and efficacy of the delivery of trainer-led AE training among participants undergoing in-person (pre–COVID-19; n=23) versus at-home telehealth AE (post–COVID-19; n=14).

**Results:**

The telehealth and in-person participants attended a similar number of exercise sessions across the 12-week interventions (26.8, SD 10.2 vs 26.1, SD 9.7, respectively; *P*=.84) and had similar number of weeks with at least 1 exercise session (10.4, SD 3.4 vs 10.6, SD 3.1, respectively; *P*=.79). The telehealth-based AE was associated with a significantly lower drop-out rate (telehealth: 0/14, 0%; in-person: 7/23, 30.4%; *P*=.04). There were no significant group differences in total time spent exercising (telehealth: 1246, SD 686 min; in-person: 1494, SD 580 min; *P*=.28); however, over the 12-week intervention, the telehealth group had a significantly lower proportion of session-time exercising at or above target intensity (telehealth: 33.3%, SD 21.4%; in-person: 63.5%, SD 16.3%; *P*<.001). There were no AE-related serious adverse events associated with either AE delivery format. Similarly, there were no significant differences in the percentage of participants experiencing minor or moderate adverse events, such as muscle soreness, joint pain, blisters, or dyspnea (telehealth: 3/14, 21%; in-person: 5/19, 26%; *P*>.99) or in the percentage of weeks per participant with at least 1 exercise-related adverse event (telehealth: 31%, SD 33%; in-person: 40%, SD 33%; *P*=.44). There were no significant differences between the telehealth versus in-person groups regarding changes in aerobic fitness as indexed by maximum oxygen consumption (VO_2_max; *P*=.27).

**Conclusions:**

Our findings provide preliminary support for the delivery of telehealth-based AE for individuals with schizophrenia. Our results indicate that in-home telehealth-based AE is feasible and safe in this population, although when available, in-person AE appears preferable given the opportunity for social interactions and the higher intensity of exercises. We discuss the findings’ clinical implications, specifically within the context of the COVID-19 pandemic, as well as review potential challenges for the implementation of telehealth-based AE among people with schizophrenia.

## Introduction

Sedentary lifestyle and low aerobic fitness are highly ubiquitous among individuals with schizophrenia [1,2] and are associated with a wide range of medical and psychiatric health indicators including cardiopulmonary and metabolic problems [3-5], high symptom burden, and depression [6], as well as poor neurocognition and daily functioning [1].

Emerging evidence over the past decade indicates that aerobic exercise (AE) training is effective in enhancing aerobic fitness in people with schizophrenia, along with improving multiple key clinical indicators [7,8]. Specifically, meta-analyses of studies of exercise in patients with schizophrenia point to significant improvements in positive, negative, and general symptoms; depressive symptoms; global functioning; quality of life (29 studies, 1109 patients) [9]; better sleep quality (8 studies, 1329 patients) [10]; as well as improved global and specific domains of neurocognition (ie, working memory, social cognition, and attention and vigilance; 10 studies, 385 patients) [7,11-13]. Aerobic exercise has also been shown to benefit daily living and social functioning (18 articles, 734 participants) [14]. Specifically, our group reported enhancements in maximum oxygen consumption (VO_2_max)–indexed aerobic fitness that significantly predicted improvements in self-, informant-, and clinician-reported social functioning, predicting 47%, 33%, and 25% of the variance, respectively [15]. Consistent with these robust findings, the European Psychiatric Association recommended AE should be incorporated into the standard treatment of people with schizophrenia and related disorders [16].

Traditionally, AE interventions for people with schizophrenia have been administered in person (eg, exercising at a gym) [17-19]. However, following the advent of the COVID-19 pandemic and the ensuing shelter-in-place regulations, many clinics transitioned their services to telehealth-based delivery. Evidence suggests that such transitions have generally been successful—a recent report of treatment engagement with mental health services among outpatients with serious mental illness (n=116,497) indicated engagement was largely maintained during the COVID-19 pandemic [20]. The utilization of telehealth-based delivery of mental health services remained high after the cancellations of the COVID-19–related shelter-in-place regulations—data from 12,828 mental health treatment facilities in the United States indicated 88% offered telehealth services in September 2022 compared with 39% in April 2019 [21]. Yet, information about the employment of telehealth-based AE among people with schizophrenia is scarce. To address this gap in the literature, our aim was to examine the feasibility, safety, and efficacy of telehealth-based delivery of AE in this population.

## Methods

### Overview

Data were obtained from a multi-site, single-blind, parallel-group, randomized clinical trial examining the impact of AE on neurocognition in individuals with schizophrenia (ClinicalTrials.gov NCT03270098). Participants were randomized to one of two 12-week, 3 times per week, 1-hour interventions: AE training or a stretching and toning (ST) control intervention. All participants received their regular medical and psychiatric treatment during the 12-week interventions. Research assessments were completed at baseline (prior to randomization) and at 6 and 12 weeks after the start of the interventions. Detailed information on the trial’s methodology has been published previously [22].

Following the onset of the COVID-19 pandemic, the interventions transitioned from an in-person format to telehealth, with identical training frequency and duration. This paper analyzes data from the AE arm of the study and compares the feasibility, safety, and efficacy of AE among participants in the in-person (pre–COVID-19) versus the telehealth AE (post–COVID-19).

### Ethical Considerations

Data were obtained from 4 US sites in New York, North Carolina, California, and Georgia. The study was reviewed and approved by the Icahn School of Medicine at Mount Sinai’s Institutional Review Board (STUDY-17‐00971); all sites approved the study, and all participants provided informed consent. Participants received up to US $720 compensation for completing all research assessments and study procedures.

### Participants

Data were collected between April 2018 and January 2023. Inclusion criteria included the following: aged 18‐55 years; English-speaking; a *Diagnostic and Statistical Manual of Mental Disorders, Fourth Edition* (*DSM-IV*) diagnosis of schizophrenia or related disorders; taking antipsychotic medication for ≥8 weeks and on current doses for ≥4 weeks or injectable depot antipsychotics for ≥3 months; medically cleared by a physician to complete a VO_2_max test and exercise; and has the capacity to understand study risks and benefits. Exclusion criteria were having a *DSM-IV* diagnosis of alcohol or substance abuse (except nicotine) within the past month or alcohol or substance dependence (except nicotine) within the past 6 months; initiation of any medication known to impact cognition in previous 4 weeks or any change in medication dosage during this period; a history of seizures or head trauma with a loss of consciousness greater than 10 minutes resulting in cognitive sequelae or rehabilitation; significant clinical abnormalities in physical examination, electrocardiogram, or laboratory assessments; medical or neurological conditions that could interfere with study participation; untreated hyper- or hypothyroidism; BMI ≥40; pregnant or nursing; serious homicidal risk within the last 6 months; “moderate” or more severe conceptual disorganization (Positive and Negative Syndrome Scale score ≥4) [23]; poor English reading ability (Wechsler Test of Adult Reading score <7) [24]; and participation in a study involving neurocognitive assessment in the previous 3 months.

### Description of In-Person and Telehealth-Based Interventions

Consistent with the American College of Sports Medicine (ACSM) guidelines [25], previous meta-analyses [26], and previous trials by our group [5,7,27,28], the AE interventions involved 12-week, 3 times per week, 1-hour exercise sessions. Sessions were led by an exercise trainer certified by the ACSM, the National Strength and Conditioning Association, or the American Council on Exercise. In-person AE sessions were held in small groups at site-affiliated exercise facilities. The in-person AE sessions began with a trainer-led 10-minute warm-up period, followed by a 45-minute exercise period using AE equipment, and ended with a 5-minute cool-down period. Participants were free to choose which AE equipment they wished to use, with the trainer encouraging participants to change equipment every 15‐20 minutes to allow all participants to use all equipment and diversify exercise routines. All sites housed identical exercise equipment (eg, a treadmill, stationary bicycle, elliptical machine, and Xbox active play video-game console with a Kinect motion-sensing device) [27,28]. Participants who missed 3 or more AE sessions due to extenuating circumstances (eg, federal holidays, inclement weather) completed an additional week of AE.

Following the COVID-19 pandemic and the ensuing shelter-in-place orders, the interventions transitioned to telehealth-based delivery. This transition was necessary due to AE involving a high degree of ventilation and the potential of aerosolization of the SARS-CoV-2 virus from infected individuals. The AE sessions were completed at the participants’ homes via teleconferencing software (CardaHealth) run on study-dedicated iPads. Telehealth AE sessions opened with a 10-minute trainer-led warm-up period, followed by 45 minutes of AE, and closed with a 5-minute cool-down period. During the 45-minute AE, participants completed common kinesthetic exercise activities (eg, jumping jacks, burpees), with a new set of exercises introduced every 3 weeks. In both AE formats, training intensity was set for each participant individually based on their maximal heart rate (HR; ie, Karvonen formula) [29] indexed during their baseline VO_2_max test, with intensity set to 60% of maximal HR in week 1, 65% in week 2, 70% in week 3, and 75% in weeks 4‐12.

AE training fidelity was indexed by the number of sessions attended and in-session training intensity recorded as the number of minutes per session participants trained with their HR at or above their designated weekly training intensity. The AE intensity was continuously monitored via Polar V800 HR monitors that participants wore during training. The trainer monitored the participant’s HR via their HR monitor, which was preprogramed to beep when the participant’s HR did not fall within the predetermined intensity window. If a participant’s HR fell under their target HR intensity level, the trainer encouraged the participant to achieve their target goal.

### Experimental Design

After fulfilling initial inclusion and exclusion criteria, participants completed a baseline assessment including demographic, diagnostic, and aerobic fitness tests (eg, VO_2_max), along with clinical, neurocognition, and daily functioning measures (not reported in this manuscript). Following completion of baseline assessments and fulfilling medical clearance (completed in the same way during the in-person and telehealth-based phases), participants were randomized consecutively in the order they entered the study to either AE or ST and began training within 1 week of randomization (in person or at home, respectively). This study makes comparisons within the AE arm between those enrolled for in-person training (prior to the COVID-19 pandemic) and those enrolled for at-home training (after the pandemic started). All baseline and 6- and 12-week follow-up research assessments (including VO_2_max tests) were completed in person during both phases of the study.

### Key Measures

#### Demographic and Clinical Measures

Demographic data were collected at the baseline assessment, along with the Structured Clinical Interview for DSM-IV Axis I Disorders (SCID-I) [30] to confirm diagnoses.

#### Aerobic Fitness

Aerobic Fitness was indexed via VO_2_max tests completed under physician supervision using electromagnetically braked cycle ergometers. Oxygen uptake and related parameters were measured at rest, during exercise, and during recovery. A 12-lead electrocardiography was used throughout all tests to index HR. Consistent with our previous studies [5, 7], the test procedure involved 3-5 minutes of resting baseline; 3-minute low resistance (10 watts) warm-up; a ramping exercise protocol of 10-50 watts (set by ACSM-predicted age, gender, and conditioning; maximal exercise norms reached in ~12 mins); and a ~3-minute active recovery period. During the VO_2_max test, workload was increased by 5-10 watts every minute until 1 of the following criteria occurred: VO_2_ plateau, 85% of maximal HR (220 beats per minute–age), a respiratory quotient ≥1.1, or reported exhaustion [31]. Pulmonary gas exchange and ventilatory variables were recorded breath-by-breath and averaged over 20 seconds.

#### AE Training Fidelity

AE training fidelity was indexed by the number of sessions a participant attended and the number of minutes per session participants trained with their HR at or above their designated weekly training intensity.

#### Safety Measures

Serious adverse events were recorded including death, hospitalization, emergency room visits, life-threatening events, disability or permanent damage, or other serious medical or psychiatric events.

Minor or moderate adverse events that are typically associated with AE were recorded weekly including blisters, chafing, fatigue, falls, muscle soreness or strain, joint pain, dyspnea, and angina.

### Statistical Analyses

Demographic and baseline clinical data were summarized for the in-person and telehealth AE groups. Total exercise sessions, weeks with exercise, total time spent exercising, and proportion of time spent at or above target exercise intensity zone per participant were summarized using means and standard deviations and compared between groups using *t* tests. All serious adverse events were listed in tables. Proportions of patients with AE-related adverse events were compared between groups using Fisher exact tests, and mean weeks or days with exercise-related events were compared with *t* tests. A mixed-effects model with the mean-centered outcome VO_2_max change from baseline was used to test for changes from baseline within each group and for differences between groups after weeks 6 and 12.

## Results

### Demographics and Sample Characteristics

One hundred and four participants enrolled in the study with 76 completing the baseline assessments and being randomized. Of the 37 randomized to the AE arm, 33 completed at least 1 exercise session (in-person: n=19; telehealth: n=14) and were included in the analyses. Table 1 presents the sample’s demographic information. There were no significant group differences with regard to primary diagnosis, age, sex, race, ethnicity, or education.

**Table 1.** Sample demographics.

Characteristic	Overall (n=33)	In person (n=19)	Telehealth (n=14)
Primary diagnosis, n (%)			
Schizophrenia	21 (64)	11 (58)	10 (71)
Schizoaffective disorder	12 (36)	8 (42)	4 (29)
Age (years), mean (SD)	37 (11)	40 (10)	33 (10)
Sex, n (%)			
Male	22 (67)	12 (63)	10 (71)
Female	11 (33)	7 (37)	4 (29)
Race, n (%)			
White or Caucasian	10 (31)	8 (44)	2 (14)
Black or African American	12 (38)	6 (33)	6 (43)
Asian, Pacific Islander, or Hawaiian	6 (19)	2 (11)	4 (29)
More than one race	4 (13)	2 (11)	2 (14)
Missing or unavailable data	1 (3)	1 (3)	0 (0)
Ethnicity, n (%)			
Hispanic or Latino	4 (12)	3 (16)	1 (7)
Non–Hispanic or Latino	29 (88)	16 (84)	13 (93)
Education, n (%)			
Did not complete high school	3 (9)	1 (5)	2 (14)
Completed high school or GED^a^	8 (24)	5 (26)	3 (21)
Some college or technical school degree	14 (42)	8 (42)	6 (43)
Completed a bachelor’s program	5 (15)	3 (16)	2 (14)
Completed graduate school	3 (9)	2 (11)	1 (7)

a GED: General Educational Development.

### Exercise Fidelity

The telehealth-based AE was associated with a significantly lower drop-out rate (telehealth: 0/14, 0%; in-person: 7/23, 30.4%; *P*=.04). However, the telehealth and in-person participants attended a similar number of exercise sessions across the 12-week interventions (26.8, SD 10.2 vs 26.1, SD 9.7, respectively; *P*=.84) and the telehealth participants also had a similar number of weeks with at least 1 exercise session (10.4, SD 3.4) compared to the in-person participants (10.6, SD 3.1; *P*=.79).

**Figure 1.**
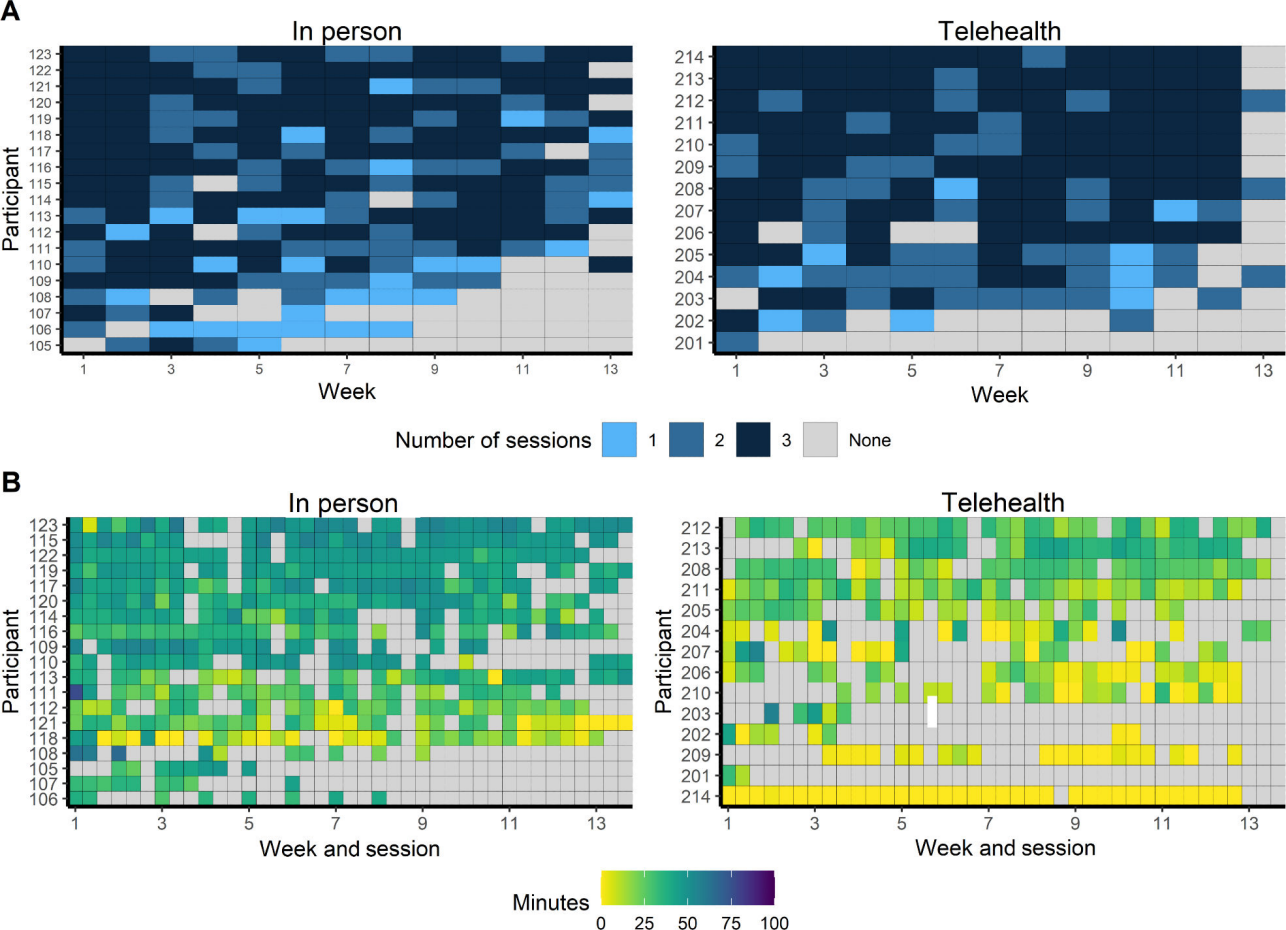
(A) Number of weekly exercise sessions and (B) session duration over the course of the intervention.

The number of weekly exercise sessions and session duration over the course of the 12-week interventions are presented in Figure 1. The telehealth participants exercised a total of 1246 (SD 686) minutes versus 1494 (SD 580) minutes among the in-person participants (*P*=.28). HR zone data were available for 96% of the exercise sessions—the in-person participants spent a significantly higher proportion of that time in the AE intensity target zone (in-person: 63.5%, SD 16.3%; telehealth: 33.3%, SD 21.4%; *P*<.001).

### Safety: Serious and Minor or Moderate Adverse Events

**Figure 2.**
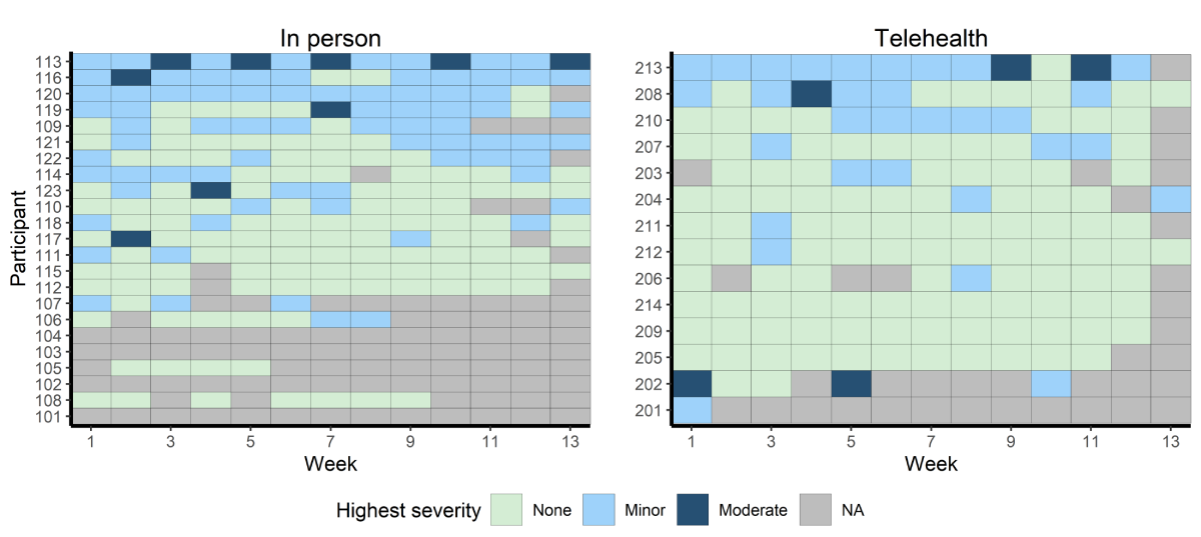
Weekly experiences of minor and moderate adverse events. NA: Not available.

None of the telehealth participants experienced any serious adverse events. The in-person AE group experienced a total of 5 serious adverse events by 2 participants (eg, exacerbation of psychotic symptoms), all resulting in psychiatric hospitalizations. All 5 adverse events were judged to be non–life-threatening and not related to the AE intervention. Data on weekly experiences of minor and moderate adverse events are presented in Figure 2.

The telehealth and in-person groups did not significantly differ in the percentage of participants with minor or moderate adverse events (telehealth: 3/14, 21%; in-person: 5/19, 26%; *P*>.99). The groups also did not differ significantly in the percentage of weeks per participant with at least 1 exercise-related adverse event (telehealth: 31%, SD 33%; in-person: 40%, SD 33%; *P*=.44) or with at least 1 moderate exercise-related adverse event (telehealth: 5%, SD 11%; in-person: 4%, SD 10%; *P*=.80).

The most common minor or moderate exercise-related adverse events experienced in at least 1 study week were soreness (n=21, 64%), fatigue (n=19, 58%), and joint pain (n=12, 36%; see Table 2). There was no significant difference between groups in the number of participants who experienced any minor or moderate exercise-related adverse events (*P*=.27). Among participants who experienced each of the minor or moderate exercise-related adverse events, the mean percentage of study days when that adverse event was experienced was highest for soreness (7.4, SD 7.4 days), fatigue (5.8, SD 6.9 days), and joint pain (3.3, SD 5.5 days), with no significant differences between groups (*P*=.15).

**Table 2.** Group comparison of experienced minor or moderate adverse events.

Characteristic	Overall (n=33), n (%)	In person (n=19), n (%)	Telehealth (n=14), n (%)	*P* value^a^
Angina	2 (6)	1 (5)	1 (7)	>.99
Blisters	1 (3)	1 (5)	0 (0)	>.99
Chafing	3 (9)	2 (11)	1 (7)	>.99
Dyspnea	6 (18)	4 (21)	2 (14)	>.99
Falls	4 (12)	2 (11)	2 (14)	>.99
Fatigue	19 (58)	12 (63)	7 (50)	.50
Joint pain	12 (36)	8 (42)	4 (29)	.49
Soreness	21 (64)	14 (74)	7 (50)	.27
Strain	7 (21)	3 (16)	4 (29)	.42

a Fisher exact test.

### Aerobic Fitness

Baseline VO_2_max was 19.1 (SD 7.25) and 21.4 (SD 7.50) for the in-person and telehealth groups, respectively. The mixed-effects model indicated an increase in VO_2_max from baseline to weeks 6 and 12 in the in-person group and a decline in the telehealth group, but these changes did not reach significance, likely due to the study being underpowered. There was no significant change from baseline to weeks 6 nor 12 in either group, nor differences between groups—the in-person AE group showed 1-to-2-point change in VO_2_max by 6 and 12 weeks that were not statistically significant. The telehealth AE group showed a 0-to-2-point decline in VO_2_max that also was not statistically significant. These changes were not different between groups at week 6 or 12 (Table 3).

**Table 3.** Group comparison of VO_2_max changes in aerobic fitness.

	In person					Telehealth					Study difference (diff)		
Timepoint	Participants, n	Raw, mean (SD)	Participants (change), n	Raw change from baseline, mean (SD)	Within-group *P* value^a^	Participants, n	Raw, mean (SD)	Participants (change), n	Raw change from baseline, mean (SD)	Within-group *P* value^a^	Study diff^b^	Cohen *d*	*P* value
Baseline	23	19.13 (7.25)	—^c^	—	—	14	21.36 (7.45)	—	—	—	—	—	—
Week 6	17	20.08 (6.75)	17	1.27 (3.38)	.32	13	19.47 (4.74)	13	–1.81 (5.51)	.18	−2.412	0.330	.10
Week 12	16	20.58 (7.51)	16	1.59 (4.55)	.13	14	20.88 (6.72)	14	−0.48 (3.52)	.91	−1.600	0.219	.27

a Within-group *P* values are obtained from longitudinal model controlling for baseline.

b Treatment effect controlling for baseline.

c Not applicable.

## Discussion

This study is the first investigation examining the feasibility, safety, and efficacy of telehealth-based AE among individuals with schizophrenia and related disorders. Our results suggest that a trainer-led administration of AE via telehealth is feasible in this population. Specifically, compared to in-person AE, telehealth-based AE was associated with a significantly lower drop-out rate and comparable total time spent exercising, though at a lower intensity as indexed by a smaller average proportion of session-time exercising at or above designated target intensity. This lower intensity may be related to the telehealth group’s exercises being limited to calisthenics versus the participants in the in-person group being able to use a variety of gym equipment, including active-play videogames [27].

Our results provide support for the safety of administering AE via telehealth, as evident by the absence of any exercise-related serious adverse events during the 12-week intervention. Likewise, compared to in-person AE, there were no significant group differences in the proportion of patients with any AE-related minor or moderate adverse events (eg, joint pain, muscle strain, blisters, dyspnea). This notable safety record can be attributed to a number of factors: all participants were screened by a physician prior to initiating AE, including a complete physical, blood work, and a VO_2_max test; attention was given to ensuring the telehealth group participants’ exercise space at their home was unobstructed and safe; for each telehealth-based exercise session, participants had to provide contact information of someone in the household in case of a medical or psychiatric emergency; the target exercise intensity was set individually for each participant based on their VO_2_max test; we employed a “start low and go slow” exercise strategy (eg, starting exercises at low intensity with gradual increases over time); a certified trainer led all training sessions and monitored participants’ training intensity via “live” HR data displayed on their screen; and weekly experiences of minor or moderate AE-related adverse events were monitored.

Somewhat surprisingly, neither group showed significant changes in aerobic fitness. This could potentially be due to the small sample size or the inclusion of a more clinically diverse patient population compared to our previous studies (eg, patients with more severe depressed mood) [7]. Additionally, the decline in VO_2_max (4.8%) in the telehealth group may reflect the impact of COVID-19 and its cardiovascular sequelae. Consistent with this view, a study of 86 nonclinical participants found a 14% decline in VO_2_max over a 3-year period coinciding with the COVID-19 pandemic (a 4.6% annual decline) [32]. Thus, it is conceivable that without the AE intervention, the telehealth participants’ aerobic fitness would have deteriorated even further. Alternatively, the lack of aerobic fitness benefits in the telehealth group may reflect having fewer opportunities to engage in physical activities outside the sessions due to the shelter-in-place guidelines. For example, an in-person AE participant who would attend 3 AE sessions per week and would walk 10 minutes to and from the gym for each AE session would engage in 60 additional minutes of physical activity per week (40% of the weekly ACSM-recommended 150 minutes of exercise). Future studies should examine the efficacy of telehealth AE during such periods without the shelter-in-place guidelines.

Telehealth-based AE presents several advantages compared to in-person AE, including (1) having a greater convenience and a potential for higher engagement; (2) allowing for AE interventions to be offered over larger geographic catchment areas, which may be particularly advantageous for delivery in rural areas or locales with limited in-person psychiatric services; (3) allowing for the delivery of AE training during periods of inclement weather that would limit travel (eg, snow storms), as well as in the event of another COVID-19–like pandemic; (4) being relatively low cost with no need for gyms, exercise equipment, or other physical infrastructure; and (5) having the digital infrastructure for its delivery already be available and in use in many hospitals and clinics, allowing it to be integrated with other clinical services (eg, use of MyChart by EPIC).

Yet, the delivery of telehealth-based AE requires consideration of some technological and logistical issues. As many individuals with schizophrenia may not have access to tablets or WiFi, we provided study participants with dedicated exercise iPads (eg, all iPad features were blocked, including internet use, email, and games). Nearly two-thirds of the devices had cellular capabilities, as most participants did not have access to home WiFi. Prior to training initiation, study personnel provided an initial introduction to iPad operations and addressed any follow-up technical or connectivity issues.

Altogether, while our findings provide preliminary support for the integration of telehealth-based AE into “real-world” clinical services for patients with schizophrenia, when available, in-person AE appears preferable given the opportunity for social interactions and the higher intensity of exercises. Additionally, the feasibility and efficacy of delivering telehealth-based AE over periods longer than 12 weeks is unknown. Thus, telehealth delivery of AE is an acceptable alternative when in-person AE is unavailable.

Our study had several limitations. First, the sample size of this study was relatively modest. Thus, any conclusions should be considered as preliminary until replicated by larger studies. Second, the delivery of AE in the present study was based in large academic medical centers with substantial medical, logistical, and other resources. Future studies should examine whether delivery in less resource-rich areas would provide comparable results. Finally, as noted, the present interventions were limited to 12 weeks, with exercise sessions completed under unique circumstances (eg, shelter-in-place orders). It is unknown whether delivery over longer periods or under normal circumstances would be similarly feasible and efficacious.

## Abbreviations

ACSM: American College of Sports Medicine
AE: aerobic exercise
*DSM-IV*: *Diagnostic and Statistical Manual of Mental Disorders, Fourth Edition*
HR: heart rate
SCID-I: Structured Clinical Interview for DSM-IV Axis I Disorders
ST: stretching and toning
VO_2_max: maximum oxygen consumption
